# Complete Genome Sequence of an Anaerobic Benzene-Degrading Bacterium, *Azoarcus* sp. Strain DN11

**DOI:** 10.1128/MRA.01699-18

**Published:** 2019-03-14

**Authors:** Allan Devanadera, Felipe Vejarano, Yu Zhai, Chiho Suzuki-Minakuchi, Yoshiyuki Ohtsubo, Masataka Tsuda, Yuki Kasai, Yoh Takahata, Kazunori Okada, Hideaki Nojiri

**Affiliations:** a Biotechnology Research Center, The University of Tokyo, Tokyo, Japan; b Collaborative Research Institute for Innovative Microbiology, The University of Tokyo, Tokyo, Japan; c Graduate School of Life Sciences, Tohoku University, Sendai, Japan; d Research and Development Initiative, Chuo University, Tokyo, Japan; e Taisei Corporation, Tokyo, Japan; University of Rochester School of Medicine and Dentistry

## Abstract

Here, we present the complete genome sequence of *Azoarcus* sp. strain DN11, a denitrifying bacterium capable of anaerobic benzene degradation. The DN11 genome is 4,956,835 bp long with a G+C content of 66.3%. Genome analysis suggested the possibility that DN11 utilizes three proposed pathways for anaerobic benzene degradation, namely, methylation, hydroxylation, and carboxylation pathways.

## Abstract (precis)

Here, we present the complete genome sequence of *Azoarcus* sp. strain DN11, a denitrifying bacterium capable of anaerobic benzene degradation.

## ANNOUNCEMENT

*Azoarcus* sp. strain DN11 (NBRC 108892) is a denitrifying, facultative anaerobic bacterium isolated from gasoline-contaminated groundwater in Kumamoto, Japan (1). DN11 can anaerobically degrade benzene and other aromatic hydrocarbons, such as toluene and *m-*xylene, under denitrifying conditions (2). These biodegradative capabilities of DN11 demonstrate its potential for bioremediation of benzene-contaminated sites under oxygen-limited conditions where benzene is persistent (3).

Three anaerobic benzene-degradation pathways have been proposed based on the metabolites identified in benzene-degrading cultures, namely, methylation to toluene, hydroxylation to phenol, and carboxylation to benzoate (4). Toluene, phenol, and benzoate are further metabolized to a common intermediate, benzoyl-coenzyme A (CoA) (4). However, enzymes involved in the three pathways have not yet been fully elucidated. Here, we report the complete genome sequence of DN11 to better understand its biochemical pathway of anaerobic benzene degradation which might involve novel genes.

DN11 was grown in diluted casitone glycerol yeast autolysate (dCGY) medium (5), and its DNA was extracted and purified using a Wizard genomic DNA purification kit (Promega). TruSeq DNA PCR-free and Nextera mate-pair library preparation kits (Illumina) were used to generate a 1,300-bp paired-end (PE) PCR-free library and a 580-bp mate-pair (MP) library with an initial fragment size of 7,100 bp. Libraries were loaded into a 600-cycle V3 chemistry sequencing cartridge, and 300 bp of each library end was sequenced with a MiSeq instrument (Illumina). All kits were used following the manufacturer’s instructions. Newbler 2.8 (Roche) was used for genome assembly (default parameters, except for overlapMinMatchIdentity of 98 and allContigThresh of 0), with 1 million PE (260 Mb) and 0.67 million MP (120 Mb) reads that were quality trimmed using ShortReadManager 0.995 (score_threshold, 30; length_threshold, 21; and 21-mers occurring more than twice regarded as valid) (6). *In silico* gap closing of the obtained scaffolds (67 in total) was done manually with AceFileViewer 1.5 and GenoFinisher 2.1 (default settings) (6) using the MP data, resulting in the complete resolution of repeat-induced gaps and unambiguous assembly of a single replicon. This sequence was checked for errors derived from the gap closing step with FinishChecker (an accessory of GenoFinisher) (7). Gene prediction and annotation were done using the Prokaryotic Genome Annotation Pipeline (PGAP) (8) and Microbial Genome Annotation Pipeline (MiGAP) (9), and annotated sequence files were first compared using GenomeMatcher 2.203 (10) to manually correct start/stop codon inconsistencies and then merged into a single annotated sequence file.

The chromosome of strain DN11 is 4,956,835 bp long (77× coverage), with a G+C content of 66.3%. Also, 4,593 coding DNA sequences (CDSs), 4 copies of the rRNA operon, and 57 tRNA genes were identified. A BLAST search of the DN11 genome revealed two *bssABCD* gene clusters involved in the anaerobic conversion of toluene to benzylsuccinate, with amino acid identities ranging from 49% to 71% compared with another anaerobic benzene-degrading bacterium, Geobacter metallireducens (11, 12). The presence of a *ppsABC* gene cluster for anaerobic conversion of phenol to phenylphosphate was identified, with 39% to 77% amino acid identities. Lastly, 3 *bamY* genes for anaerobic conversion of benzoate to benzoyl-CoA had 44%, 49%, and 50% amino acid identities. They indicated the possibility that to degrade benzene under anaerobic conditions, DN11 may use all three pathways, namely, methylation, carboxylation, and hydroxylation pathways.

### Data availability.

The complete genome sequence of *Azoarcus* sp. strain DN11 has been deposited at DDBJ/ENA/GenBank under the accession number CP021731 (software parameters and details of the use and rationale of bioinformatics tools for the completion of the genome sequence are available in the “comment” section). Raw sequencing data have been deposited in the SRA under the accession number PRJNA388763.
